# An unusual association of left‐sided gastroschisis and persistent right umbilical vein

**DOI:** 10.1002/ccr3.1897

**Published:** 2018-11-05

**Authors:** Ingrid Anne Mandy Schierz, Giuseppa Pinello, Mario Giuffrè, Giovanni Corsello

**Affiliations:** ^1^ Neonatal Intensive Care Unit, A.U.O.P. “P. Giaccone”, Department of Sciences for Health Promotion and Mother and Child Care “G. D’Alessandro” University of Palermo Palermo Italy

**Keywords:** abdominal wall defect, gastrointestinal malformation, prenatal diagnosis

## Abstract

Gastroschisis is a full‐thickness congenital abdominal wall defect usually occurring to the right of the umbilicus. About twenty cases of left‐sided gastroschisis have been reported, without reference to the laterality of the umbilical vein. This first case highlights the importance of considering and reporting this association by the perinatal team.

A 1650 g male neonate was born at 33 + 3 weeks gestation by cesarean to a 31‐year‐old mother. Prenatal ultrasonography (Figure 1) detected left‐sided gastroschisis, persistent right umbilical vein (PRUV), and right aortic arch. Molecular karyotyping resulted normal.

**Figure 1:**
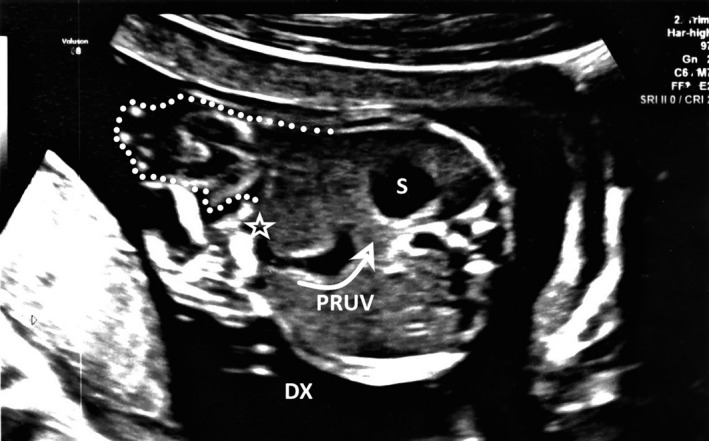
Left‐sided herniation of dilated bowel (dotted line) and persistent right umbilical vein (PRUV) turning toward the stomach (S) were first detected at 21 wk gestation and monitored with serial fetal transverse abdominal US

At birth, a 2.5 cm wide abdominal wall defect was identified to the left of the umbilical cord (Figure 2). Stomach, small and large bowel were eviscerated and thickened. They were not malrotated, atresic or stenotic, but with a Meckel diverticulum. Imaging investigations did not reveal situs abnormalities.

**Figure 2:**
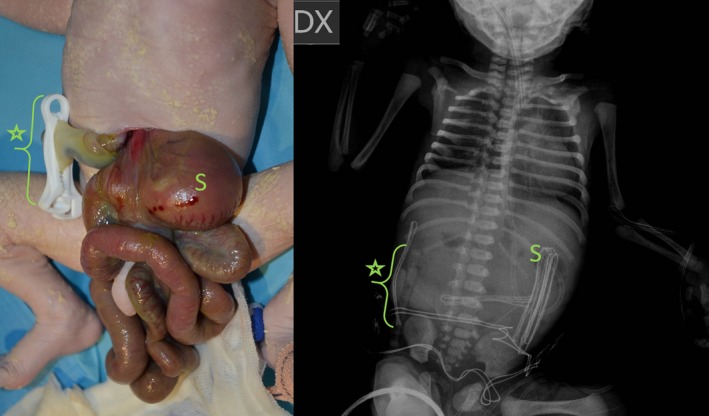
Gastroschisis placed on the left of the umbilical cord (asterisk); the X‐ray confirmed a levocardia and a normally left‐located stomach (S)

After 24 hours of the primary repair, the patient developed an abdominal compartment syndrome. Despite of exteriorization of the bowel by silo, he expired at 36 hours.

Gastroschisis, unlike omphalocele, is not commonly associated with chromosomal disorders and extra‐intestinal anomalies.^1^ However, left‐sided gastroschisis is associated with other anomalies, and can be a laterality defect indicator.^1^, ^2^ Our case, although presenting a right aortic arch, does not meet diagnostic criteria for heterotaxy.

The underlying pathogenetic mechanism of gastroschisis is still unclear. The most reliable mechanism is timely abnormal resorption of the right umbilical vein during embryogenesis.^1^ This case suggests a mirror theory of PRUV and concomitant regression of the left umbilical vein as a cause of left‐sided gastroschisis.

## CONFLICT OF INTEREST

None declared.

## AUTHOR CONTRIBUTION

IAMS: involved in conception, writing, and revision of the manuscript. GP: involved in acquisition of data and revised literature. MG and GC: revised the manuscript for intellectual content.
